# Effects of Exercise and Nutritional Intervention on Body Composition, Metabolic Health, and Physical Performance in Adults with Sarcopenic Obesity: A Meta-Analysis

**DOI:** 10.3390/nu11092163

**Published:** 2019-09-09

**Authors:** Kuo-Jen Hsu, Chun-De Liao, Mei-Wun Tsai, Chiao-Nan Chen

**Affiliations:** 1 Department of Physical Therapy and Assistive Technology, School of Biomedical Science and Engineering, National Yang Ming University, Taipei 11221, Taiwan; 2 Department of Physical Medicine and Rehabilitation, Shuang Ho Hospital, Taipei Medical University, New Taipei City 23561, Taiwan

**Keywords:** resistance exercise, aerobic exercise, supplementation

## Abstract

People with sarcopenic obesity (SO) are characterized by both low muscle mass (sarcopenia) and high body fat (obesity); they have greater risks of metabolic diseases and physical disability than people with sarcopenia or obesity alone. Exercise and nutrition have been reported to be effective for both obesity and sarcopenia management. Thus, we aimed to investigate the effects of exercise and nutrition on body composition, metabolic health, and physical performance in individuals with SO. Studies investigating the effects of exercise and nutrition on body composition, metabolic health, and physical performance in SO individuals were searched from electronic databases up to April 2019. Fifteen studies were included in the meta-analysis. Aerobic exercise decreased body weight and fat mass (FM). Resistance exercise (RE) decreased FM and improved grip strength. The combination of aerobic exercise and RE decreased FM and improved walking speed. Nutritional intervention, especially low-calorie high-protein (LCHP) diet, decreased FM but did not affect muscle mass and grip strength. In addition to exercise training, nutrition did not provide extra benefits in outcome. Exercise, especially RE, is essential to improve body composition and physical performance in individuals with SO. Nutritional intervention with LCHP decreases FM but does not improve physical performance.

## 1. Introduction

Sarcopenia involves progressive and generalized loss of muscle mass that is associated with physical disability, metabolic dysfunction, and increased mortality [1,2,3]. Obesity is a risk factor of many chronic diseases including cardiovascular (CVD) and metabolic diseases [4]. Obesity also impairs muscle quality and decreases physical ability [5,6]. Sarcopenic obesity (SO) is the term used to describe the condition involving both low muscle mass (sarcopenia) and high body fat (obesity) [7]. Compared to individuals who only have sarcopenia or obesity, individuals with SO have greater risks of metabolic disorders, higher CVD prevalence, higher mortality rates, and reduced physical performance, such as walking speed [8,9,10,11,12]. To manage obesity, many strategies have been proposed including lifestyle intervention (exercise and nutrition), pharmacologic therapy, and bariatric surgery [13]. Among these, exercise and nutrition are also key interventions for sarcopenia management [14,15]. Regarding SO management, to date, only a few systematic reviews and meta-analysis studies have provided information about the effects of exercise and nutritional intervention [16,17,18]. Two systemic reviews where limited number of studies were eligible for examination (two and eight studies, respectively) showed that exercise alone but not protein or amino acid supplementation alone improved muscle strength and physical function of individuals with SO [16,17]. Regarding the effects on muscle mass, current reviews found the diversity of the results of studies and the conclusion could not be made [16,17]. By meta-analysis, Hita-Contreras et al., showed that exercise training alone or combined with protein supplementation increases muscle mass, grip strength, and walking speed in individuals with SO [18]. Nevertheless, effects of different types of exercise have not been examined separately and that outcome measures of metabolic parameters have not yet been investigated in the study of Hita-Contreras et al. Metabolic health is associated with lower prevalence rates of CVD and metabolic diseases. In addition, identifying the effective type of exercise is critical to translate this knowledge into clinical practice for weight management and improving physical function in the SO population. Therefore, the aims of this study were (1) to investigate the effects of exercise or nutrition on body composition, metabolic health, and physical performance in individuals with SO and (2) to determine if exercise combined with nutrition provides additional benefits for body composition, metabolic health, and physical performance, compared to exercise or nutrition alone, in individuals with SO.

## 2. Materials and Methods 

This meta-analysis was performed according to the Preferred Reporting Items for Systematic Reviews and Meta-Analyses (PRISMA) guidelines [19].

### 2.1. Search Strategies

We used PubMed, Scopus, Clinical Key, and Cochrane Library without language or publication date restrictions. A systematic search was performed for eligible studies published up to April 2019. The keywords were (“sarcopenia” OR “sarcopenic”) AND (“obese” OR “obesity” OR “obestic” OR “overweight”) AND (“exercise” OR “training” OR “physical” OR “nutrition” OR “restriction” OR “diet” OR “supplementation”) (Table S1).

### 2.2. Study Selection

Studies were included if they met the following criteria: (1) clinical trial designs, (2) written in English and or were non-English articles translated into English, (3) participants had SO, (4) interventions consisted of exercise, a diet program, or a combination of both, (5) outcome measurements included one of the following: body composition, metabolic or inflammatory biomarkers, muscle function, or physical performance. Studies were excluded if they met the following criteria: (1) were observational, secondary data or animal studies, (2) the comparisons were not performed according to the intervention type.

### 2.3. Data Extraction

Two authors independently extracted the data from the selected studies into a Microsoft Excel spreadsheet; any disagreement was resolved in a consensus meeting. Table 1 presented the summary of the extracted data where the following information was presented: (1) authors, publication year, and country, (2) study design, (3) characteristics of the study population (sample size and demographics), (4) definition of SO, (5) types of intervention, (6) duration of intervention, and (7) outcome findings for body composition, metabolic health, muscle function, and physical function. One author extracted data and another author checked the extracted data.

### 2.4. Quality Assessment

Two authors independently completed an assessment of the methodological quality of each included study using the Physiotherapy Evidence Database (PEDro) scale. If the score was inconsistent between two authors (Hsu KJ, Chen CN), a third author was consulted (Tsai MW) to judge the final score. The PEDro scale has 10 items: (1) random allocation, (2) concealed allocation, (3) similarly at baseline, (4) participants blinding, (5) therapists blinding, (6) assessors blinding, (7) drop-out rate <15%, (8) intention to treat analysis, (9) between group statistical analysis, and (10) point and variability measurement; the score ranges from 0 to 10. A score < 4 indicates a low-quality study. The score ≥ 6 indicates a high-quality study [35].

### 2.5. Statistical Analysis

Statistical analysis was performed using Review Manager 5.3. (The Nordic Cochrane Centre, Copenhagen, Denmark) Means, standard deviations, and the sample size of extracted data were input to the statistic software. Extracted variables were converted to the same unit and mean differences (MDs) were compared between the intervention and control groups. Analyses were stratified according to type of exercise (e.g., aerobic [AE] vs. control; resistance [RE] vs. control; and combined exercise [CE] vs. control) and the type of nutrition (e.g., supplementation vs. control; low-calorie high protein [LCHP] vs. control). In all analyses, *I*^2^ statistics were used to analyze heterogeneity between studies. A random-effects model was used if the *p*-value of heterogeneity test (*I*^2^ statistic) was <0.05; otherwise, the fixed-effects model was used. 

## 3. Results

### 3.1. Study Selection

**Figure 1:**
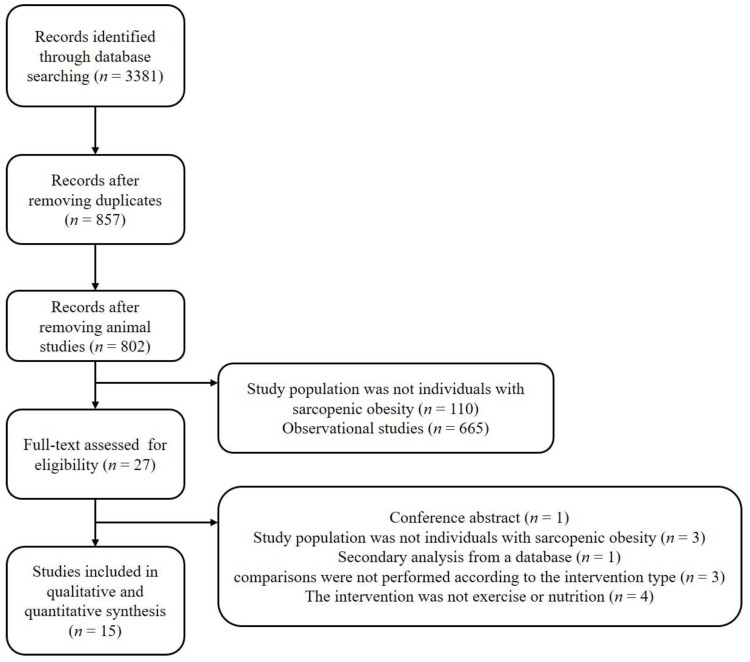
The study selection process.

Based on the search key words, 3381 original studies were initially identified from the databases. After removing duplicates and animal studies, the titles and abstracts of 802 studies were screened. After the screening, the full texts of 27 studies were assessed. From these, one study was excluded as it was a conference abstract; three were excluded as the study population did not consist of individuals with SO; four studies were excluded as the subjects did not undergo exercise or nutritional interventions; three studies were excluded as the comparisons were not performed according to the intervention type; and one study was excluded as it was a secondary analysis from a database. Fifteen studies were included in the current meta-analysis; among which, 14 were randomized controlled trials [20,21,22,24,25,26,27,28,29,30,31,32,33,34] and one was designed as a quasi-experimental trial [23] (Figure 1).

### 3.2. Study Characteristics

Among the 15 included studies, only 10 recruited women as subjects [20,23,24,26,27,28,30,31,32,33,34]; another two only recruited men as subjects [25,29]; the remaining three recruited both sexes [21,22,23]. The age of subjects among the included studies ranged from 41 to 90 years old; the average age of subjects in 13 studies was ≥65 years old. In terms of the definition used for of obesity, six studies used BMI (the cutoff point was 25 kg/m^2^ in two studies and 30 kg/m^2^ in the others) [21,22,29,30,32,34] and nine studies used body fat percentage (BF%) (men >27–30%, women >30–40%) [20,23,24,25,26,27,28,31,33]. Regarding the definition of sarcopenia, skeletal muscle mass (appendicular skeletal mass (ASM), ASM/squared body height (Ht^2^), ASM/body weight (BW), ASM/BMI, total skeletal mass (TSM)/Ht^2^, TSM/BW, or ideal fat-free mass (FFM) was used in 14 studies [20,21,22,23,24,25,26,27,28,29,30,31,32,33], and grip strength was used in one study [34] (Table 1).

### 3.3. Exercise Protocol

Nine studies investigated the effects of exercise on SO [21,22,23,24,26,27,28,32,34]. Among them, one compared the effects among the AE, RE, CE, and control groups [22]; five studies compared the effects between the RE and control groups [23,24,27,28,34]; two studies compared the effects between the CE and control groups [26,32]; and one study compared the effects between the RE and power training groups [21]. Following were the training periods in the included studies: two studies, less than 12 weeks [22,34]; five studies, 12 weeks [23,24,26,27,28]; and one study, 24 weeks [32]. In terms of exercise intensity and duration, AE was set at moderate intensity; the duration was 40–45 minutes [22]. Intensity of RE was set at moderate intensity [21,22,23,24,27,28,34]; moderate intensity was defined as 13 on the rating of perceived exertion scale in four studies [23,24,27,28], and it was defined as 40%–75% of one-repetition maximum (1RM) in three studies [21,22,34]. Intensity of power training was set at the maximal power outputs [21]. For the studies investigating the effects of RE, a single study focused on lower extremity muscle groups [34], and the others focused on the major muscle groups of the whole body [22,23,24,27,28]. Three studies investigated the effects of CE (the combination of AE and RE) where the intensity of AE was set at moderate to high and the duration was 12–50 minutes; RE was performed on the key muscle groups of the whole body [22,26,32] (Table S2).

### 3.4. Protocol of Nutritional Intervention

Five studies investigated the effects of nutrition on SO [20,25,26,30,33]. Among these, three studies compared the effects between the supplementation and control groups [20,25,26]; two studies compared the effects between LCHP and low-calorie normal protein diet (LCNP) [30,33]. For studies that examined the effects of supplementation, one study involved an intervention with isoflavone 70 mg/day for 6 months [20]; one involved intervention with protein supplementation (1.7−1.8 g/kg BW/day) for 4 months [25]; and one involved intervention with essential amino acid supplementation together with catechin-fortified tea for 3 months [26]. In studies comparing the effects between LCHP and LCNP, high protein was defined as 1.2 g/kg/day, and low calorie was defined as 20–25 kcal intake/kg/day [30] and 90% of daily energy requirement (metabolic rate) [33] (Table S3).

### 3.5. Protocols of Interventions that Combined Exercise and Nutrition 

Three studies investigated the additional effects of supplementation on SO subjects with exercise training [26,29,31]. One study examined the additional effects of 4 months of dairy and non-dairy isocaloric and isoprotein supplementation than RE alone [29]. Another study examined the additional effects of three months of supplementation (essential amino acid and tea fortified with catechins) than CE alone [26]. The other study examined the additional effects of 4-month protein supplementation than RE alone [31] (Tables S2 and S3).

### 3.6. Risk of Bias in Included Studies

The PEDro score was presented in Table 1. The average PEDro score of the enrolled studies was 6.6 ± 1.5. Among these, eleven studies were high-quality studies [23,24,25,27,29,30,31,34,35]; the others were medium quality studies [21,22,24,30,32,33]. All included studies had between group comparisons and both point and variability measurements. In addition, 14 studies had random allocation; four had subject blinding; three had therapist blinding; 13 had assessor blinding; 11 had adequate follow-up; four used intention to treat analysis (Table S4). 

### 3.7. Effects of Exercise on Body Composition

There were seven studies investigating the effects of exercise on body composition [22,23,24,26,27,28,32]. Five used bioelectrical impedance analysis (BIA) and two used dual-energy X-ray absorptiometry (DXA) to determine body composition. 

**Figure 2:**
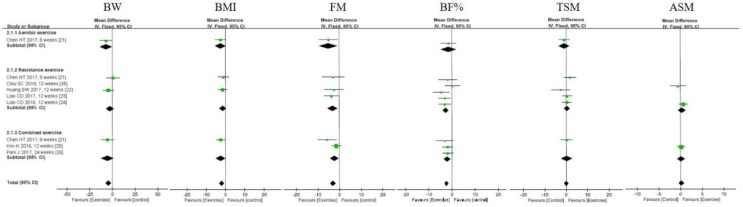
Forest plots of comparisons between exercise and the control groups for parameters of body composition in individuals with sarcopenic obesity. ASM: appendicular skeletal muscle mass; BF%: body fat percentage; BMI: body mass index; BW: body weight; FM: total fat mass; TSM: total skeletal muscle mass.

Overall, exercise decreased BW (MD = −4.3 kg, 95% CI: −7.61, −0.99, *p* = 0.01; *I*^2^ = 0%), BMI (MD = −1.98 kg/m^2^, 95% CI: −3.29, −0.67, *p* = 0.003, *I*^2^ = 0%), body fat mass (FM) (MD = −2.99 kg, 95% CI: −4.39, −1.59, *p* < 0.0001, *I*^2^ = 0%), and BF% (MD = −2.31%, 95% CI: −3.26, −1.36, *p* = 0.00001, *I*^2^ = 0%). Exercise training did not have significant effects on ASM and TSM. Subgroup analysis based on exercise type (i.e., RE, AE, and CE) showed that (1) BW only decreased by AE (MD = −7.1 kg, 95% CI: −13.87, −0.33, *p* = 0.04) and (2) BF% only decreased by RE (MD = −2.67%, 95% CI: −4.03, −1.32, *p* = 0.0001; *I*^2^ = 17%, *p* > 0.05) and CE (MD = −2.05%, 95% CI: −3.5, −0.61, *p* = 0.005; *I*^2^ = 0%) (Figure 2 and Figure S1).

### 3.8. Effects of Exercise on Metabolic and Inflammatory Biomarkers

**Figure 3:**
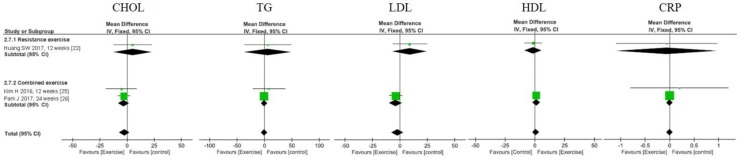
Forest plots of comparisons between exercise and the control groups for lipid profiles and C-reactive protein (CRP) in individuals with sarcopenic obesity. CHOL: total cholesterol; HDL: high density lipoprotein; LDL: low density lipoprotein; TG: triglycerides.

Three studies investigated the effects of exercise on metabolic and inflammatory biomarkers [24,26,32]. Exercise, regardless of type, did not improve metabolic and inflammatory biomarkers, including total cholesterol (CHOL), triglycerides (TG), low density lipoprotein, high density lipoprotein (HDL), and C-reactive protein (CRP) (Figure 3 and Figure S2). 

### 3.9. Effects of Exercise on Muscle Strength and Walking Speed

**Figure 4:**
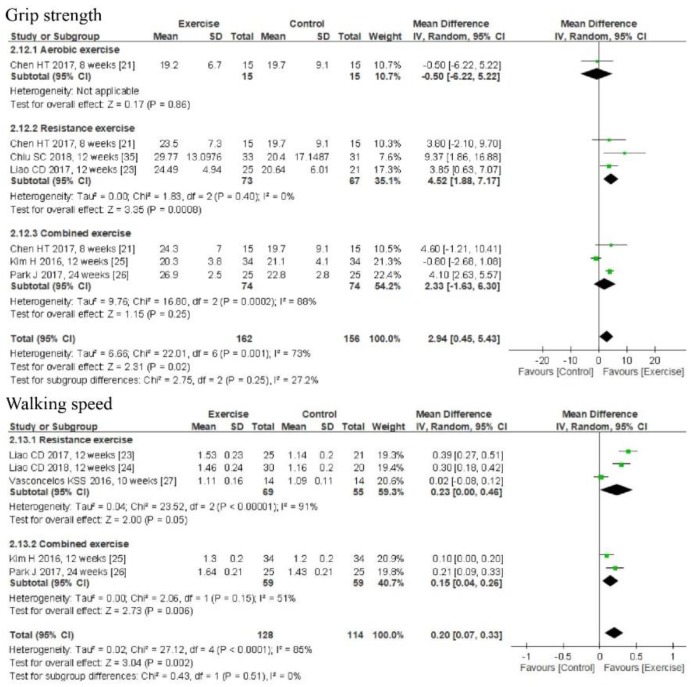
Forest plots of comparisons between exercise and the control groups on grip strength and walking speed in individuals with sarcopenic obesity.

Five studies investigated the effects of exercise on grip strength [22,23,26,27,32]. Meta-analysis results indicated that exercise increased grip strength (MD = 2.94 kg, 95% CI: 0.45–5.43, *p* = 0.02; *I*^2^ = 73%, *p* = 0.001); however, a subgroup analysis found this effect only with RE (MD = 4.52 kg, 95% CI: 1.88–7.17, *p* = 0.0008; *I*^2^ = 0%) [22,23,27]. In contrast, AE and CE did not improve grip strength [22,26,32]. Five studies investigated the effects of exercise on walking speed, and the pooled results showed that exercise increased walking speed (MD = 0.2 m/s, 95% CI: 0.07–0.33, *p* = 0.002; *I*^2^ = 85, *p* < 0.0001) [26,27,28,32,34]. Subgroup analysis found that walking speed increased after CE (MD = 0.15 m/s, 95% CI: 0.04–0.26, *p* = 0.006; *I*^2^ = 51%, *p* > 0.05) (Figure 4).

### 3.10. Effects of Nutritional Intervention on Body Composition

**Figure 5:**
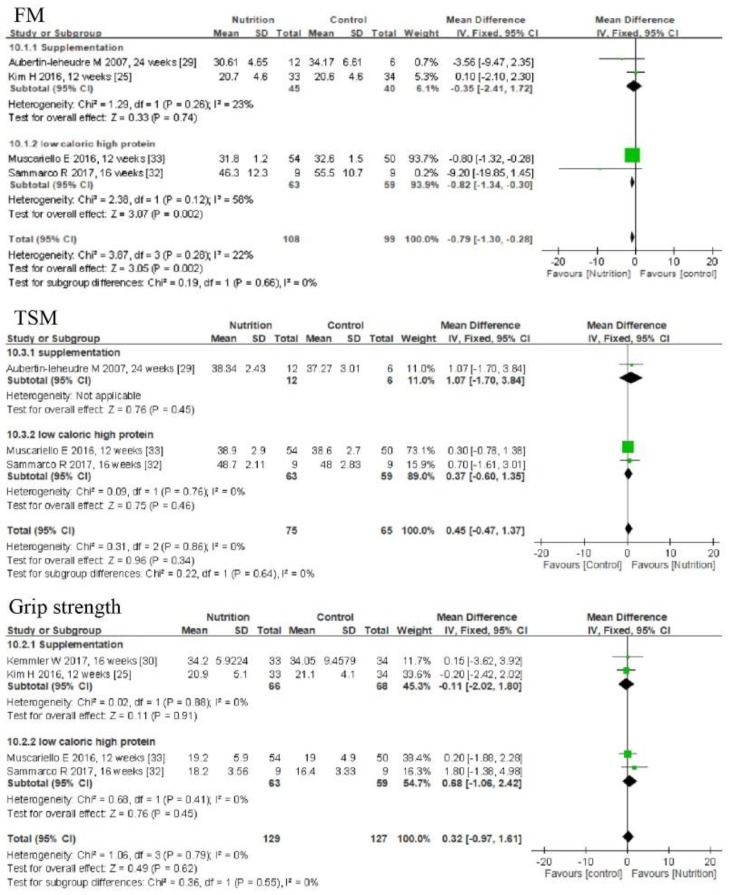
Forest plots of comparisons between nutrition and the control groups for total fat mass (FM), total skeletal muscle mass (TSM), and grip strength in individuals with sarcopenic obesity.

Four studies investigated the effects of nutrition on body composition, including FM and TSM [20,26,30,33]. Among these, three used BIA and one used DXA to determine body composition. The meta-analysis results indicated that nutritional intervention decreased FM but did not improve muscle mass, compared to that in the control groups (MD = −0.79 kg, 95% CI: −1.3, −0.28, *p* = 0.002; *I*^2^ = 0%). A subgroup analysis found that only LCHP decreased FM more than that in the control group (MD = −0.82 kg, 95% CI: −1.34, −0.3, *p* = 0.002; *I*^2^=58%, *p* > 0.05); however, supplementation did not decrease FM (Figure 5). 

### 3.11. Effects of Nutrition on Muscle Strength

Four studies investigated the effects of nutrition on grip strength [25,26,30,33]. The meta-analysis results indicated that nutritional intervention did not improve grip strength (MD = 0.32 kg, 95% CI: −0.97, 1.61; *I*^2^ = 0%) (Figure 5). 

### 3.12. Additional Effects of Nutritional Supplementation on Exercise Training

Three studies investigated the additional effects of protein-based supplementation during exercise training on body composition and on metabolic and inflammatory biomarkers [26,29,31]. Among these, one used BIA and two used DXA to determine body composition. The results showed that protein supplementation in addition to exercise training, did not have extra effects on body composition, or on metabolic and inflammatory biomarkers (Figures S3–S5).

## 4. Discussion

This study aimed to investigate the effects of exercise and nutrition on body composition, metabolic health, and physical performance in individuals with SO. We found that exercise intervention (8–24 weeks) improved body composition in terms of BW, BMI, FM, BF%, grip strength, and walking speed in the SO population. Nutritional interventions (12–24 weeks) decreased FM with no changes in grip strength. Markers of metabolism and inflammation in individuals with SO were not changed followings exercise interventions. Compared to exercise or nutritional intervention alone, interventions that combined exercise and nutrition did not confer additional beneficial effects on body composition, grip strength, walking speed, or metabolic and inflammatory biomarkers in the SO population. Results from the subgroup analysis based on exercise type (i.e., RE, AE, and CE) showed that: (1) BW decreased only by AE; (2) BF% decreased more by RE and CE than by AE; (3) grip strength increased only with RE; and (4) walking speed increased with CE. In the sub-analysis based on the type of nutritional intervention (supplementation vs. LCHP), FM decreased with LCHP but not supplementation. Both supplementation and LCHP were ineffective in improving muscle mass and grip strength in individuals with SO.

The goals of SO interventions are (1) to lose FM, (2) to improve fat-associated dysregulation of metabolism and inflammation, (3) to increase muscle mass, and (4) to improve muscle strength and physical function. Exercise and nutrition are the two key interventions in the management of SO. It has been shown that exercise alone or combined with supplementation decreased FM and increased TSM in elderly individuals with SO [18]. Consistent with this previous finding, our study found that exercise decreased FM. By further examining the differential effects of types of exercises on body composition and physical performance, we found that in the SO population, RE improves physical performance while both AE and RE decreased FM. The explanation for the preferential effects of RE on physical performance is that obesity exacerbates the physical capacity of individuals with sarcopenia [36], and since RE is the optimal exercise type to increase muscle strength [37], physical performance (e.g., grip strength and walking speed) significantly improves in SO individuals following RE. 

Surprisingly, the current study did not find greater TSM in SO individuals who underwent an exercise intervention compared to the control group. This finding is different from that described by Hita-Contreras et al.; they found that exercise increased TSM in SO individuals. The inconsistent results between our study and the study by Hita-Contreras et al. are due to differences in study selection criteria. Here, studies with interventions which included active muscle contractions were included. Hita-Contreras et al., included studies with interventions consisting of both active and passive muscle contractions (e.g., exercise and whole-body electrical stimulation). The ES intervention contributed to the increase in TSM in the meta-analysis by Hita-Contreras et al. Collectively, 8–24 weeks of exercise did not significantly increase TSM in individuals with SO. Nevertheless, exercise prevented age-related muscle loss in individuals with SO. For instance, studies reported that while TSM did not change after exercise intervention, it significantly decreased in the control groups [24,27].

CVD morbidity and mortality are greater in individuals with SO. Therefore, for the SO population, we should not only focus on body composition and physical performance but also on metabolic health and inflammatory biomarkers. We found that exercise did not improve lipid profile and CRP. However, previous studies have shown that exercise improved lipid profile in adults [38,39], especially HDL and TG. The differences in findings between our study and those previously published are likely due to three reasons. First, the number of studies examining the effects of exercise and nutritional interventions on metabolic health and inflammation is very limited (3 RCTs, total 153 subjects). Previous meta-analyses in which data were pooled from 41–49 RCTs (total 1715–2990 individuals) found significant effects of exercise on lipid profiles [40,41]. Second, the intervention duration and exercise volume were relatively short in most of the included studies. Only one study performed exercise training for 24 weeks where they showed exercise decreased CHOL level [32]. In contract, studies with exercise training for 12 weeks did not find any changes in metabolic and inflammatory biomarkers [24,26]. Regarding exercise intensity, it was shown that high intensity exercise provides more benefits than moderate intensity exercise [39]. However, enrolled studies in the study that investigated the effects of exercise on metabolic and inflammatory markers utilized moderate intensity exercise [24,26,32]. Lastly, all eligible studies excluded individuals with chronic diseases. It was shown that exercise training improves lipid profile in adults with dyslipidemia; however, it does not change lipid profiles in adults without dyslipidemia [39]. The present study showed that nutritional intervention, especially LCHP, decreased FM. However, nutritional intervention did not increase TSM and muscle strength in the SO population. Low caloric intake (LC) is an effective way to reduce BW and FM in the obesity population. However, LC also decreases TSM [42]. In the current study, we found that LC combined with high protein (HP) decreased FM and preserved TSM. HP (without LC) is a suggested nutrition strategy for increasing TSM and muscle strength in the sarcopenic population [43,44]. Importantly, we found that nutritional intervention alone did not improve grip strength in the SO population. Muscle strength is determined by many factors, such as neuromuscular control and muscle mass. This finding suggests that exercise is the foundation for the increase in muscle strength and physical performance. For instance, the study by Kim et al. reported that while the stride length of gait did not increase in the SO population after nutritional intervention, it increased in individuals after the combined intervention with nutrition and exercise [26]. Taken together, nutritional intervention with LCHP improves body composition but not physical performance in the SO population. 

The present study showed that nutrition did not provide additional benefits on exercise training for the SO population. For all the included studies, the nutritional strategy was protein supplementation immediately after exercise [26,29,31]. Previous studies showed that protein supplementation provided additional beneficial effects on TSM and muscle strength compared to exercise alone in older adults [45,46]. In contrast, in the sarcopenia population, protein supplementation did not provide additional effects on TSM and physical performance than exercise alone [47]. In the current study where the SO population was investigated, only one included study reported additional beneficial effects of protein supplementation on TSM and FM vs. exercise alone [31]. Other studies found no additional effects [26,29]. The inconsistent results may due to different definitions of SO. While the concept of SO is clear (i.e., individuals with the condition involving both sarcopenia and obesity), the operational definition is inconsistent. For instance, some studies defined obesity according to BMI [21,22,29,30,32,34] while others defined obesity by BF% [20,23,24,25,26,27,28,33]. Some studies defined sarcopenia by skeletal muscle mass [20,21,22,23,24,25,26,27,28,29,30,32,33], some studies defined it by muscle strength [26,34] and others defined it by physical performance [26]. Future research establishing universally accepted cut-offs for SO is required to enhance the awareness and the advancement of SO research.

There are some limitations in the study. First, only a few studies investigated the effects of exercise or nutrition on metabolic and inflammatory biomarkers in the SO population. Since SO is associated with the highest prevalence of CVD, compared to sarcopenia or obesity alone, more studies investigating the effects of interventions on metabolic and inflammatory biomarkers are needed. Second, there was only one study that examined the effects of AE; thus, we could not conclude the effects of AE on body composition, metabolic health, and physical performance in individuals with SO. Third, there was only one study examining the additional effects of exercise (plus nutrition) vs. nutrition alone on the SO population; thus, the associated conclusion could not be made. Finally, the operational definitions of SO were inconsistent among included studies.

## 5. Conclusions

Exercise, especially RE, is essential to improve body composition and physical performance in individuals with SO. Nutritional intervention with LCHP can decrease FM but may not improve physical performance. Protein supplementation did not provide additional benefits on body composition or on metabolic and inflammatory biomarkers, compared to exercise alone, in the SO population. 

## Figures and Tables

**Table 1:** Characteristics of studies.

Study (Country) [ref]	Study Design	Groups (Sample Size)	Age (Years)	Sex	Definition of Sarcopenic Obesity		Assessment Tool of Body Composition	Time Point of Measurement	Results	PEDro Score
					Sarcopenia	Obesity				
**Aubertin-Leheudre M., 2007 (Canada) [20]**	RCT	Isoflavones (12) Control (6)	58.0 ± 5.0	Women	SMI (ASM/Ht^2^) <6.87 kg/m^2^	BF% >40	DXA	Baseline: 0 week Posttest: 24 weeks	Legs FFM, ASM and SMI ↑ BW, BMI, WC, FM, TSM ⇌	8
**Balachandran A., 2014 (US) [21]**	RCT	RE (9) PT (8)	71.3 ± 8.0	Both	SMI (TSM/Ht^2^) <10.76 kg/m^2^ in men; <6.76 kg/m^2^ in women	BMI >30 kg/m^2^	BIA	Baseline: 0 week Posttest: 15 weeks	leg press power (PT) ↑ SPPB, 1RM of leg press and chest press ⇌	4
**Chen H.T., 2017 (Taiwan) [22]**	RCT	AE (15) RE (15) CE (15) Control (15)	68.8 ± 3.3	Both	SMI (ASM/BW) ≤32.5% in men; ≤25.7% in women	BMI ≥25 kg/m^2^ and VFA ≥100 cm^2^	BIA	Baseline: 0 week Posttest: 8 weeks Follow up: 12 weeks	BW (AE and CE), BMI (CE), TSM, SMI, FM, BF%, VFA and BES (AE, RE and CE), GS (RE), KES (RE and CE) ↑	5
**Chiu S.C., 2018 (Taiwan) [23]**	Quasi-experimental study	RE (33) Control (31)	79.9 ± 7.8	Both	SMI (TSM/BW) <37.15% in men; <32.26% in women	BF% >29 in men BF% >40 in women	BIA	Baseline: 0 week Posttest: 12 weeks	GS, PS ↑ ASM, BF%, SMI ⇌	4
**Huang S.W., 2017 (Taiwan) [24]**	RCT	RE (18) Control (17)	69.2 ± 5.0	Women	SMI (TSM/BW) <27.6%	BF% >30	DXA	Baseline: 0 week Posttest: 12 weeks	FM, BF% ↑ BW, BMI, TSM, SMI, TG, HDL, LDL, CHOL, CRP ⇌	7
**Kemmler W., 2017 (Germany) [25]**	RCT	Protein (33) Control (34)	77.5 ± 5.1	Men	SMI (ASM/BMI) <0.789	BF% >27	BIA	Baseline: 0 week Posttest: 16 weeks	BF%, SMI ↑ GS ⇌	8
**Kim H., 2016 (Japan) [26]**	RCT	EN (36) E (35) N (34) Control (34)	81.1 ± 4.6	Women	SMI (ASM/Ht^2^) <5.67kg/m^2^ or GS <17 kg or WS <1m/s	BF% >32	BIA	Baseline: 0 week Posttest: 12 weeks	FM (EN), TFM, stride and step length (E) ↑ BF%, ASM, SMI, GS, KES, WS, SBP, CHOL, TG, CRP, IL-6 and HBA1c ⇌	6
**Liao C.D., 2017 (Taiwan) [27]**	RCT	RE (25) Control (21)	67.3 ± 5.1	Women	SMI (TSM/Ht^2^) <7.15 kg/m^2^	BF% >30	DXA	Baseline: 0 week Posttest: 12 weeks	TSM, FM, BF%, SLS, WS, TUG, TCR, GS ↑	8
**Liao C.D., 2018 (Taiwan) [28]**	RCT	RE (30) Control (20)	67.3 ± 5.1	Women	SMI (TSM/BW) <27.6%	BF% >30	BIA	Baseline: 0 week Posttest: 12 weeks Follow up: 36 weeks	BF%, TSM, ASM, SMI, SLS, WS, TUG, TCR ↑	9
**Maltais M.L., 2016 (Canada) [29]**	RCT	RE + Nondairy shake (8) RE+Dairy shake (8) RE (10)	65.2 ± 4.8	Men	SMI (ASM/Ht^2^) <10.75 kg/m^2^	BMI >30 kg/m^2^	DXA	Baseline: 0 week Posttest: 16 weeks	FM (RE+Dairy shake and RE+Nondairy shake) ↑ BMI, TSM, GLU, insulin, CHOL, TG, HDL, LDL, leptin, TNF-a, IL-6 and CRP ⇌	6
**Muscariello E., 2016 (Italy) [30]**	RCT	LCNP (50) LCHP (54)	66.7 ± 4.9	Women	SMI (TSM/Ht^2^) <7.3 kg/m^2^	BMI >30 kg/m^2^	BIA	Baseline: 0 week Posttest: 12 weeks	BMI, WC, FM, TSM, SMI, GS ⇌	6
**Nabuco H.C.G., 2019 (Brazil) [31]**	RCT	RE + protein (13) RE (13)	69.0 ± 4.1	Women	ASM <15.02 kg	BF% >35	DXA	Baseline: 0 week Posttest: 16 weeks	TSM, ASM, FM, BF% and IL-6 (RE+protein) ↑ WC, CHOL, TG, HDL, LDL, GLU, insulin, HOMA-IR, TNF-a, CRP, SBP ⇌	8
**Park J., 2017 (Korea) [32]**	RCT	CE (25) Control (25)	74.1 ± 6.1	Women	SMI (ASM/BW) <25.1%	BMI >25 kg/m^2^	BIA	Baseline: 0 week Posttest: 24 weeks	WC, BF%, GS, TCR, SBP, CHOL, LDL ↑ ASM, TG, HDL, CRP ⇌	7
**Sammarco R., 2017 (Italy) [33]**	RCT	LCNP (9) LCHP (9)	55.0 ± 9.6	Women	<90% of ideal FFM^a^	BF% >34.8	BIA	Baseline: 0 week Posttest: 16 week	TSM (LCHP) ↑ BW, FM, BF%, GS, SPPB ⇌	5
**Vasconcelos K.S., 2016 (Brazil) [34]**	RCT	RE (14) Control (14)	72.0 ± 4.1	Women	GS ≤21 kg	BMI ≥30 kg/m^2^	NA	Baseline: 0 week Posttest: 10 week	WS, SPPB, KES, KEP ⇌	8

AE: aerobic exercise; ASM: appendicular muscle mass; BIA: bioelectrical impedance analysis; BES: back extensor strength; BF%: body fat percentage; BMI: body mass index; BW: body weight; CE: combined exercise; CHOL: total cholesterol; CRP: C-reactive protein; DXA: dual-energy X-ray absorptiometry; E: exercise; EN: exercise plus nutrition; FFM: fat-free mass; FM: total fat mass; GLU: glucose; GS: grip strength; HbA1c: Glycated hemoglobin; HDL: high density lipoprotein; Ht: body height; HOMA-IR: Homeostatic model assessment of insulin resistance; IL-6: Interleukin 6; KEP: knee extensor power; KES: knee extensor strength; LCHP: low-calorie high-protein; LCNP: low-calorie normal-protein; LDL: low density lipoprotein; NA: the related information was not provided in the manuscript; PEDro: Physiotherapy Evidence Database; PS: pinch strength; PT: power training; RCT: randomized controlled trial; RE: resistance exercise; SBP: systolic blood pressure; SLS: single leg stance; SMI: skeletal muscle index; SPPB: short physical performance battery; TCR: timed chair rise; TFM: trunk fat mass; TG: triglycerides; TNF-a: tumor necrosis factor alpha; TSM: total skeletal muscle; TUG: timed up and go; VFA: visceral fat area; WC: waist circumference; WS: walking speed; 1RM: one-repetition maximum. a: Ideal FFM is calculated by 0.75 × ideal BW + 0.25 × excess BW; ↑: improved; ⇌; no change

## Supplementary Materials

The following are available online at https://www.mdpi.com/2072-6643/11/9/2163/s1, Figure S1: Forest plots of comparisons between exercise and the control group for the parameters of body composition in individuals with sarcopenic obesity. ASM: appendicular skeletal muscle mass; BF%: body fat percentage; BMI: body mass index; BW: body weight; FM: total fat mass; TSM: total skeletal muscle mass, Figure S2: Forest plots of comparisons between exercise and the control group for lipid profiles and C-reactive protein (CRP) in individuals with sarcopenic obesity. CHOL: total cholesterol; HDL: high density lipoprotein; LDL: low density lipoprotein; TG: triglycerides. Figure S3. Forest plots of comparisons between exercise plus nutrition (EN) and exercise alone (E) for the parameters of body composition in individuals with sarcopenic obesity, Figure S4: Forest plots of comparisons between exercise plus nutrition (EN) and exercise alone (E) for the parameters of metabolic health in individuals with sarcopenic obesity. HDL: high density lipoprotein; LDL: low density lipoprotein, Figure S5: Forest plots of comparisons between exercise plus nutrition (EN) and exercise alone (E) for inflammatory biomarkers in individuals with sarcopenic obesity. IL-6: Interlukin-6; TNF-a: Tumor necrosis factor-alpha; CRP: C-reactive protein, Table S1: Databases search process, Table S2: Exercise protocols, Table S3: Protocols of nutritional intervention, Table S4: The PEDro score of included studies.
