# Supplementary material for: Effects of Exercise and Nutritional Intervention on Body Composition, Metabolic Health, and Physical Performance in Adults with Sarcopenic Obesity: A Meta-Analysis
Source: Nutrients. 2019 Sep 9;11(9):2163. doi: 10.3390/nu11092163 (PMC6770949; doi:10.3390/nu11092163)
Supplement: Supplementary file 1 [file nutrients-11-02163-s001.pdf]

Table 1. Databases search process.

Databases	PubMed, Scopus, Clinical key and Cochrane Library
Steps	Search terms for query
#1	“Sarcopenia” OR “sarcopenic”
#2	“obese” OR “obesity” OR “obestic” OR “overweight”
#3	“exercise” OR “training” OR “physical”
#4	“nutrition” OR “restriction” OR “diet” OR “supplementation”
#5	“#1” AND “#2” AND “#3”
#6	“#1” AND “#2” AND “#4”
#7	“#1” AND “#2” AND “#3” AND “#4”

Table 2. Exercise protocols.

Study	Exercise types	Intensity	Duration	Frequency (times/wk)	Period (weeks)
Chen HT., 2017	AE: Aerobic dance	Moderate intensity	60 minutes	2	8
Balachandran A., 2014	RE: Whole body major muscle groups	70% of 1RM	10–12 repetitions, 3 sets	2	15
Chen HT., 2017	RE: Whole body major muscle groups	60-70% of 1RM	8-12 repetitions, 3 sets	2	8
Chiu SC., 2018	RE: Whole body major muscle groups	2-5 lbs	4-10 repetitions, 3 sets	2	12
Huang SW., 2017	RE: Whole body major muscle groups	13 points on the RPE scale	10 repetitions, 3 sets	3	12
Liao CD., 2017	RE: Whole body major muscle groups	13 points on the RPE scale	10 repetitions, 3 sets	3	12
Liao CD., 2018	RE: Whole body major muscle groups	13 points on the RPE scale	10 repetitions, 3 sets	3	12
Maltasis ML., 2016	RE: Whole body major muscle groups	80% of 1RM	8 repetitions, 3 sets	3	16
Nabuco HCG., 2019	RE: Whole body major muscle groups	70% of 1RM	8-12 repetitions, 3 sets	3	12
Vasconcelos KSS., 2016	RE: Lower extremity muscle groups	50-75% of 1RM	8-12 repetitions, 2-3 sets	2	10
Chen HT., 2017	CE: AE (dance steps)<br>RE (whole body major muscle groups)	AE: Moderate intensity<br>RE: 60~70% of 1RM	AE: 60 minutes<br>RE: 8-12 repetitions, 3 sets	AE: 1<br>RE: 1	8
Kim H., 2016	CE: AE (stationary bicycle)<br>RE (whole body major muscle groups)	AE: up to 40 watts<br>RE: NA	AE: 12 minutes<br>RE: 10 repetitions, 3 sets	AE: 2<br>RE: 2	12
Park J., 2017	CE: AE (walking activities)<br>RE (whole body major muscle groups)	AE: 13-17 points on the RPE scale<br>RE: NA	AE: 30-50 minutes<br>RE: 8-15 repetitions, 2-3 sets	AE: 5<br>RE: 3	24
Balachandran A., 2014	PT: Whole body major muscle group	Maximal power outputs	10–12 repetitions, 3 sets	2	15

AE: aerobic exercise; CE: combined exercise; NA: not available; PT: power training; RE: resistance exercise; RPE: rating of perceived exertion; 1RM: one repetition maximum.

Table 3. Protocols of nutritional intervention.

Study	Type of Intervention	Description of Interventions
Aubertin-Leheudre M., 2007	Supplementation	Isoflavone (70 mg/day)
Kim H., 2016	Supplementation	AA (3 g/day) and catechin (540 mg /day)
Kemmler W., 2017	Supplementation	Protein supplementation (1.7-1.8 g/BW(kg)/day) and vitamin D (800 IU/day)
Maltasis ML., 2016	Supplementation	Protein shake (12 g) immediate after exercise
Nabuco HCG., 2019	Supplementation	Hydrolyzed whey protein (35 g) immediate after exercise
Muscariello E., 2016	LCHP	LC (20-25 kcal/IBW(kg)/day and HP (1.2 g/IBW(kg)/day)
Sammarco R., 2017	LCHP	LC (90% of daily energy requirement) and HP (1.2-1.4 g/BW(kg)/day)
Muscariello E., 2016	LCNP	LC (20-25 kcal/IBW(kg)/day and NP (0.8 g/IBW(kg)/day)
Sammarco R., 2017	LCNP	LC (90% of daily energy requirement) and NP (0.8-1.0 g/BW(kg)/day)

AA: amino acid; BW: body weight; HP: high protein; IBW: ideal body weight; LC: low calorie; LCHP: low calorie plus high protein; LCNP: low calorie plus normal protein; NP: normal protein.

Table 4. The PEDro score of included studies.

Study	Eligibility Criteria Were Specified	(1)	(2)	(3)	(4)	(5)	(6)	(7)	(8)	(9)	(10)	Total
Aubertin-Leheudre, M., 2007	Y	Y	Y	Y	Y	Y	Y	N	N	Y	Y	8
Balachandran, A., 2014	Y	Y	N	N	N	N	Y	N	N	Y	Y	4
Chen, H. T., 2017	Y	Y	N	Y	N	N	Y	N	N	Y	Y	5
Chiu, S. C., 2018	Y	N	N	Y	N	N	Y	N	N	Y	Y	4
Huang, S. W., 2017	Y	Y	Y	Y	N	N	Y	Y	N	Y	Y	7
Kemmler, W., 2017	Y	Y	Y	Y	N	N	Y	Y	Y	Y	Y	8
Kim, H., 2016	Y	Y	Y	Y	N	N	N	Y	N	Y	Y	6
Liao, C. D., 2017	Y	Y	N	Y	N	Y	Y	Y	Y	Y	Y	8
Liao, C. D., 2018	Y	Y	Y	Y	N	Y	Y	Y	Y	Y	Y	9
Maltais, M. L., 2016	Y	Y	N	N	Y	N	Y	Y	N	Y	Y	6
Muscariello, E., 2016	Y	Y	N	Y	N	N	Y	Y	N	Y	Y	6
Nabuco H. C. G., 2019	Y	Y	Y	Y	Y	N	Y	Y	N	Y	Y	8
Park, J., 2017	Y	Y	N	Y	Y	N	Y	Y	N	Y	Y	7
Sammarco, R., 2017	Y	Y	N	Y	N	N	N	Y	N	Y	Y	5
Vasconcelos, K. S., 2016	Y	Y	Y	Y	N	N	Y	Y	Y	Y	Y	8

(1) random allocation, (2) concealed allocation, (3) similar at baseline, (4) participants blinding, (5) therapists blinding, (6) assessors blinding,. (7) adequate follow up, (8) intention to treat analysis, (9) between group statistic analysis, (10) point and variability measurement.

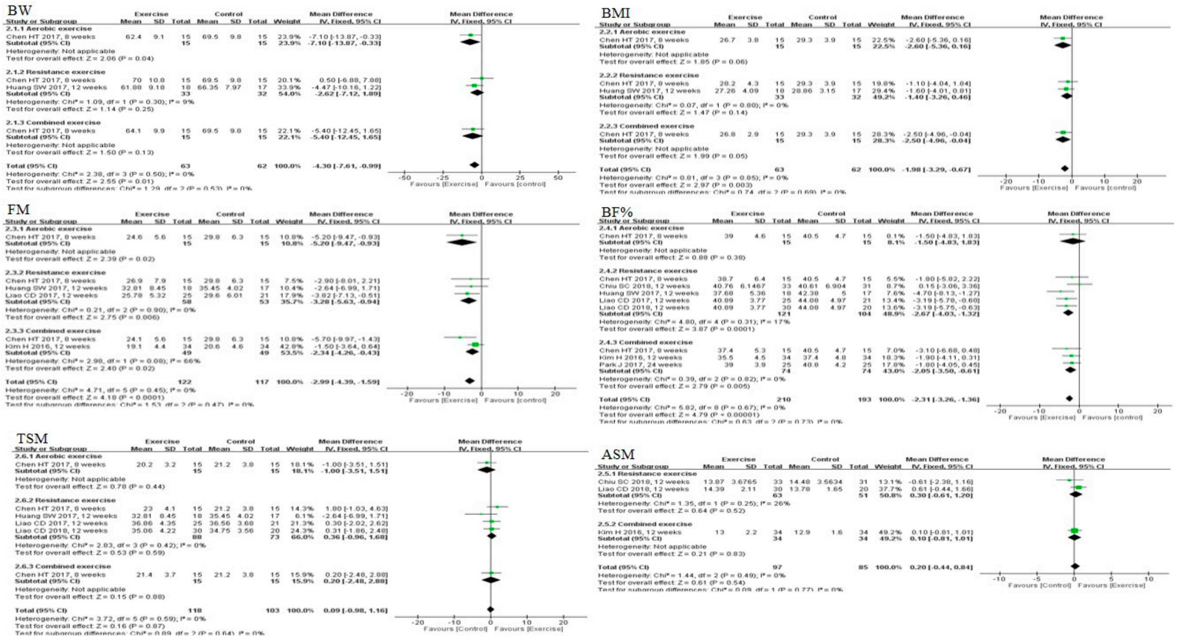

Figure 1. Forest plots of comparisons between exercise and the control group for the parameters of body composition in individuals with sarcopenic obesity. ASM: appendicular skeletal muscle mass; BF%: body fat percentage; BMI: body mass index; BW: body weight; FM: total fat mass; TSM: total skeletal muscle mass.

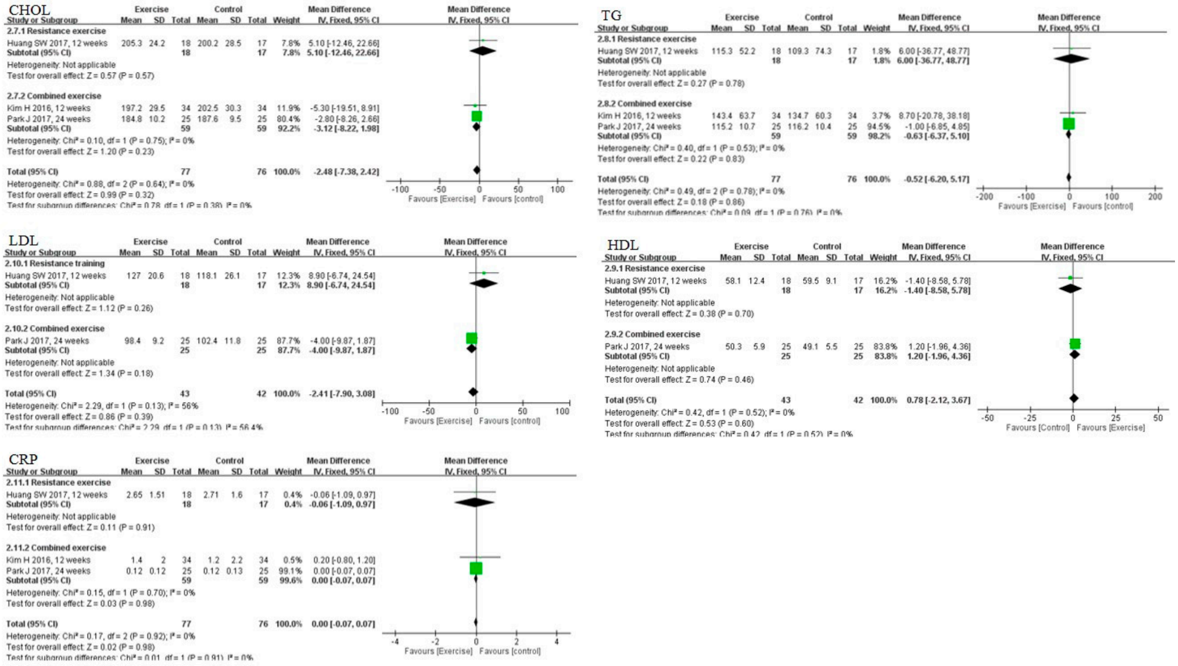

Figure 2. Forest plots of comparisons between exercise and the control group for lipid profiles and C-reactive protein (CRP) in individuals with sarcopenic obesity. CHOL: total cholesterol; HDL: high density lipoprotein; LDL: low density lipoprotein; TG: triglycerides.

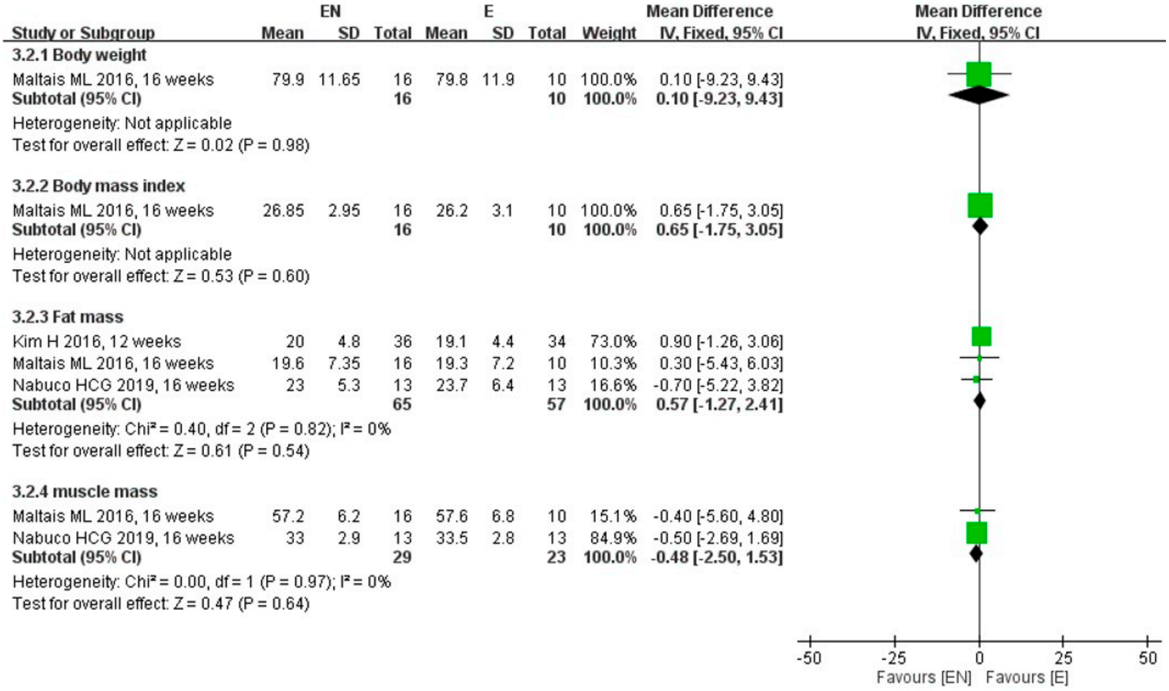

Figure 3. Forest plots of comparisons between exercise plus nutrition (EN) and exercise alone (E) for the parameters of body composition in individuals with sarcopenic obesity.

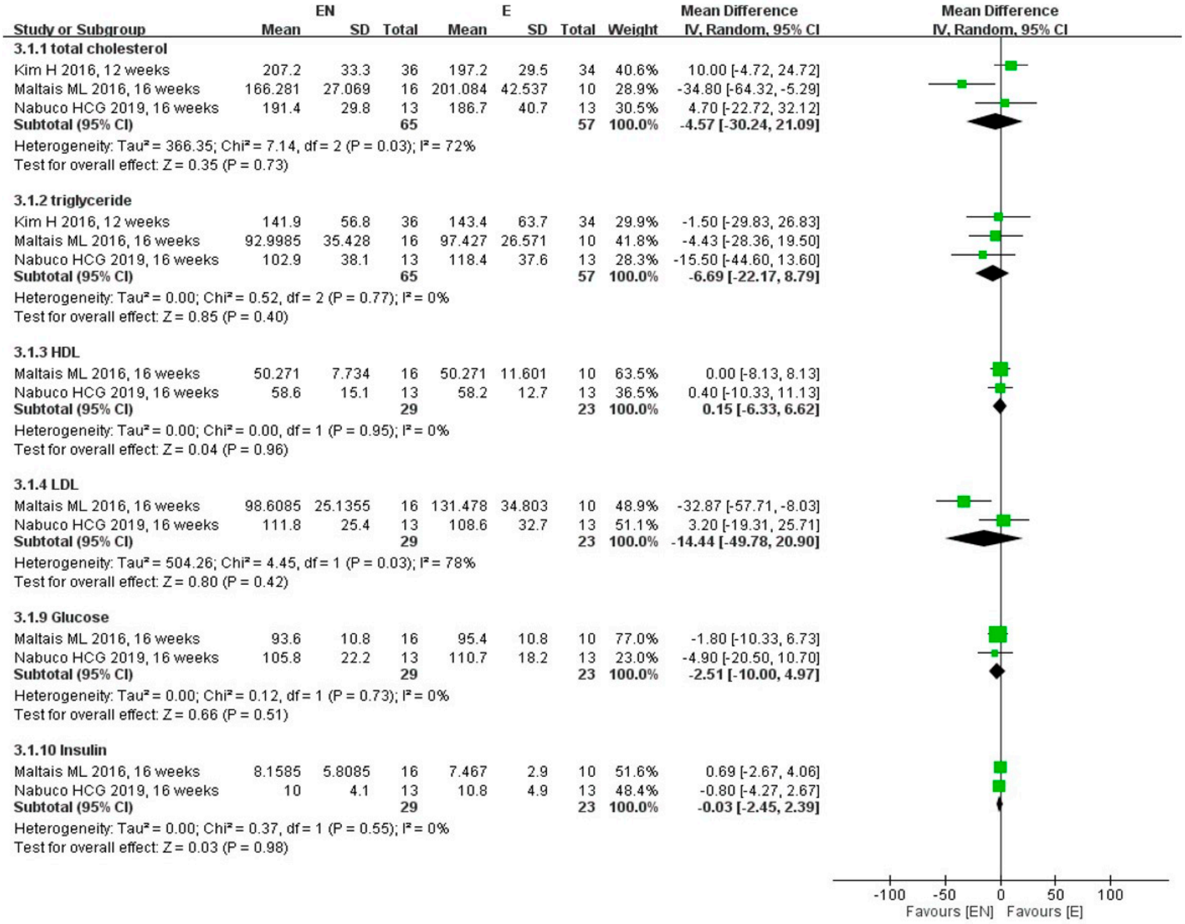

Figure 4. Forest plots of comparisons between exercise plus nutrition (EN) and exercise alone (E) for the parameters of metabolic health in individuals with sarcopenic obesity. HDL: high density lipoprotein; LDL: low density lipoprotein.

Study or Subgroup	EN Mean	EN SD	EN Total	E Mean	E SD	E Total	Weight	Mean Difference IV, Fixed, 95% CI
**3.3.2 IL-6**								
Kim H 2016, 12 weeks	3.7	4.2	36	3.7	2.3	34	6.2%	0.00 [-1.57, 1.57]
Maltais ML 2016, 16 weeks	1.245	0.545	16	1.43	0.58	10	76.1%	-0.18 [-0.63, 0.26]
Nabuco HCG 2019, 16 weeks	2.1	0.6	13	2.9	1.6	13	17.7%	-0.80 [-1.73, 0.13]
**Subtotal (95% CI)**			**65**			**57**	**100.0%**	**-0.28 [-0.67, 0.11]**
Heterogeneity: Chi² = 1.50, df = 2 (P = 0.47); I² = 0%								
Test for overall effect: Z = 1.42 (P = 0.16)								
**3.3.3 TNF-a**								
Maltais ML 2016, 16 weeks	1.605	0.4	16	1.69	0.32	10	98.0%	-0.08 [-0.36, 0.19]
Nabuco HCG 2019, 16 weeks	2.3	1.6	13	3.4	3.2	13	2.0%	-1.10 [-3.04, 0.84]
**Subtotal (95% CI)**			**29**			**23**	**100.0%**	**-0.11 [-0.38, 0.17]**
Heterogeneity: Chi² = 1.03, df = 1 (P = 0.31); I² = 2%								
Test for overall effect: Z = 0.75 (P = 0.45)								
**3.3.4 CRP**								
Kim H 2016, 12 weeks	1.3	2.4	36	1.4	2	34	44.1%	-0.10 [-1.13, 0.93]
Maltais ML 2016, 16 weeks	1.285	0.96	16	1.81	2.01	10	26.5%	-0.53 [-1.86, 0.81]
Nabuco HCG 2019, 16 weeks	2.2	1	13	2.2	2.1	13	29.4%	0.00 [-1.26, 1.26]
**Subtotal (95% CI)**			**65**			**57**	**100.0%**	**-0.18 [-0.87, 0.50]**
Heterogeneity: Chi² = 0.36, df = 2 (P = 0.84); I² = 0%								
Test for overall effect: Z = 0.52 (P = 0.60)								

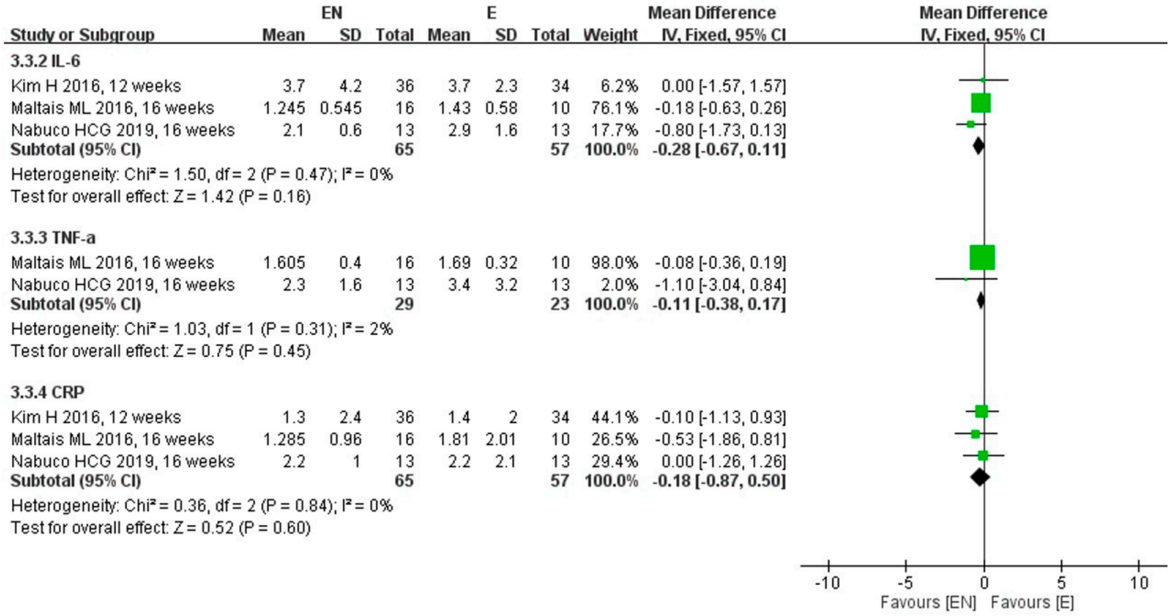

Figure S5. Forest plots of comparisons between exercise plus nutrition (EN) and exercise alone (E) for inflammatory biomarkers in individuals with sarcopenic obesity. IL-6: Interlukin-6; TNF-a: Tumor necrosis factor-alpha; CRP: C-reactive protein.
